# Nitrogen-Containing Apigenin Analogs: Preparation and Biological Activity

**DOI:** 10.3390/molecules171214748

**Published:** 2012-12-11

**Authors:** Rui Liu, Bin Zhao, Dong-En Wang, Tianyu Yao, Long Pang, Qin Tu, Saeed Mahmoud Ahmed, Jian-Jun Liu, Jinyi Wang

**Affiliations:** 1 Colleges of Science and Forestry, Northwest A&F University, Yangling 712100, Shaanxi, China; 2 College of Agronomy and Life Sciences, Shanxi Datong University, Datong 037009, Shanxi, China

**Keywords:** apigenin analogs, antiproliferative activity, antibacterial activity, antioxidant activity

## Abstract

A series of nitrogen-containing apigenin analogs **4a**–**j** was synthesized via Mannich reactions to develop anticancer, antibacterial, and antioxidant agents from plant-derived flavonoids. The chemical structures of these compounds were confirmed using ^1^H-NMR, ^13^C-NMR, and ESI-MS. The *in vitro* biological activities of the analogs were evaluated via assays of their antiproliferative, antibacterial, and antioxidant activities. The prepared apigenin analogs exhibited different antiproliferative activities against four human cancer cell lines, namely human cervical (HeLa), human hepatocellular liver (HepG2), human lung (A549), and human breast (MCF-7) cancer cells. Compound **4i** showed the most favorable *in vitro* antiproliferative activity with IC_50_ values of 40, 40, 223, and 166 μg/mL against HeLa, HepG2, A549, and MCF-7, respectively. The 1,1-diphenyl-2-picrylhydrazyl (DPPH) free radical scavenging activity assay also showed that **4i** had the most potent antioxidant activity, with the smallest IC_50_ value (334.8 μg/mL). The antibacterial activities of the analogs were determined using a two-fold serial dilution technique against four pathogenic bacteria, namely *Staphylococcus aureus*, *Bacillus subtilis*, *Escherichia coli*, and *Pseudomonas aeruginosa*. All the prepared apigenin analogs exhibited more potent activities than the parent apigenin. Compounds **4h** and **4j**, in particular, exhibited the best inhibitory activities against the Gram-positive bacteria *Staphylococcus aureus* and *Bacillus subtilis* with MIC values of 3.91 and 1.95 μg/mL, respectively.

## 1. Introduction

Flavonoids are important components of various traditional Chinese medicines and phytomedicines that bear C_6_-C_3_-C_6_ skeletons [1,2]. In addition to their physiological roles in plants, these compounds also exhibit anticancer, antioxidant, anti-aging, and antibacterial activities [3,4,5]. Flavonoids display antibacterial activity by inhibiting nucleic acid synthesis [6,7,8], cytoplasmic membrane function [9,10,11], and energy metabolism [12]. They are generally classified as flavones, flavonols, flavanones, flavanonol, or isoflavones, based on the degree of oxidation of the carbon-3 bond (C-3) and the connection position of the B-ring [13]. Flavonoids are prevalent in higher plants as well as in the roots, stems, leaves, and flowers of ferns [14]. To date, over 4,000 flavonoids have been identified, and several of these compounds showed diverse pharmacological activities, such as antimicrobial [15], antiallergic [16], antidiabetic [17], anti-inflammatory [18], antiviral [19], antimutagenic [20], antithrombotic [21], antioxidant [22], anticarcinogenic [23], and hepatoprotective [24] effects.

Apigenin [2-(4-hydroxyphenyl)-5,7-dihydroxy-4*H*-chromen-4-one], which belongs to the flavone subclass of flavonoids, are found in fruits, vegetables, and traditional medicinal plants, such as parsley, onion, orange, tea, chamomile, wheat sprouts, and several seasonings [25]. Apigenin exhibits anti-inflammatory and anti-carcinogenic effects on the skin [26]. In some cells, apigenin displays a variety of anti-tumor effects, such as stimulation of gap junctional and intracellular communication [27] as well as inhibition of mutagenesis [28], transformation [29], angiogenesis [30], and tumorigenesis [31]. In addition, apigenin exhibits antibacterial activity against a number of bacterial species [32,33]. Previous studies have shown the potential of apigenin as a bioactive molecule for various clinical applications. However, compared with existing drugs, the anticancer, antibacterial, antioxidant, and other activities of apigenin hardly meet the requirements for clinical application. Considerable effort has thus been exerted to improve the activities of apigenin [34,35,36,37]. A simple and effective way is the study of the structure-activity relationships of apigenin analogs prepared by introducing various functional groups into different positions in the apigenin skeleton [38,39,40]. Gunnarsson *et al.* [40] synthesized sulfated apigenin analogs, which were all antithrombin activators to accelerate the inhibition of factor Xa. Mavel *et al*. [38] synthesized apigenin analogs with halogen on the 4'-position which showed cytotoxic activity and MDR-reversing capacity. However, all the current studies mainly focused on the 4'- or 7-position of apigenin. The 8-position of apigenin was scarcely examined.

The Mannich reaction is a classic method for the preparation of Mannich bases, namely, *β*-amino ketones and *β*-amino aldehydes. The Mannich reaction is also one of the very important reaction types and key reaction-steps in the synthesis of numerous drugs and natural products [41,42]. This reaction has been considered as an effective method in introducing aminomethyl substituents into the desired molecules to improve their biological activities [43]. Previous studies have demonstrated that Mannich bases offer a wide range of biological activities, such as anticancer [44], anti-inflammatory [45], analgesic [46], antibacterial, and antifungal activities [47]. The inhibitory activities of adriamycin salicylamide Mannich base against MCF-7 and PC-3 cells are four times better than that of adriamycin, while simultaneously reducing the clinical side effects [48]. In addition, several studies have shown that 8-aminomethylated oroxylin A analogs demonstrated significantly improved α-glucosidase inhibitory activity [49], nitrogen-containing baicalein/quercetin analogs exhibited potent CDK1/cyclin B inhibitory activity [50], and 8-aminomethylluteolin derivatives displayed potential anti-inflammatory activity [51].

Thus, in the present study, a series of new apigenin analogs with aminomethyl groups on the C-8 position was synthesized via Mannich reactions. In addition, the antiproliferative, antibacterial, and antioxidant activities of these analogs were investigated. The antiproliferative activities of the compounds were analyzed against four human cancer cell lines, namely the human cervical (HeLa), human hepatocellular liver (HepG2), human lung (A549), and human breast (MCF-7) cancer cells, using a standard 3-(4,5-dimethylthiazol-2-diphenyl-tetrazolium) bromide (MTT) assay. The antibacterial activities of the prepared apigenin analogs against four pathogenic bacteria, namely *Staphylococcus aureus* (*S. aureus*), *Bacillus subtilis* (*B. subtilis*), *Escherichia coli* (*E. coli*), and *Pseudomonas aeruginosa* (*P. aeruginosa*), were explored using a two-fold serial dilution technique. The antioxidant activities of the apigenin analogs were determined using the DPPH radical scavenging assay.

## 2. Results and Discussion

### 2.1. Chemistry

**Scheme 1:**
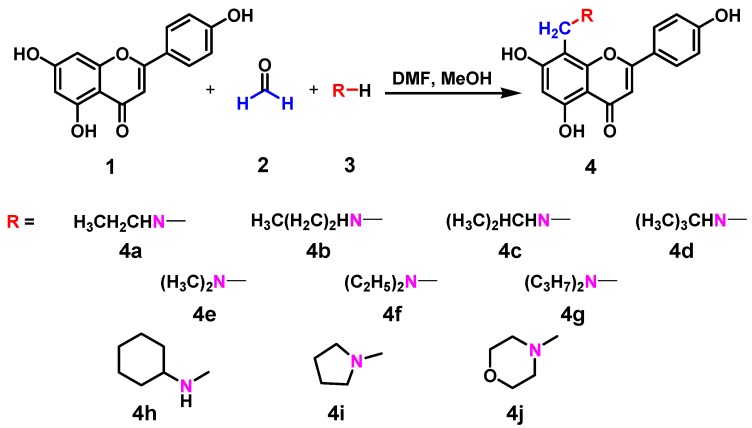
Synthetic route of compounds **4a** to **4j**.

Ten new nitrogen-containing apigenin analogs, **4a**–**j**, were prepared via Mannich reactions (Scheme 1) to further explore their antiproliferative, antibacterial, and antioxidant activities. The hydrogen atom activity of apigenin in the C-8 position increases because of the p-π conjugative effect of the 5- and 7- hydroxy in the A-ring. Thus, apigenin easily reacts with formaldehyde and primary or secondary amines to produce different Mannich bases [52]. The reaction conditions, yields, and structures of the apigenin analogs are summarized in Table 1. Corresponding products were obtained with the introduction of different primary or secondary amines. The reaction temperatures had a little difference for different products, varied from 25 °C to 40 °C. Also, different times were required for these reactions since no or less byproducts and better yields were obtained under these conditions.

**Table 1:** Physicochemical data of the nitrogen-containing apigenin analogs.

Compounds	R	Time (h)	Temperature (°C)	Isolated yield (%)
**4a**	Ethylamino	4	25	40.5
**4b**	Propylamino	12	30	38.7
**4c**	Isopropylamino	4	35	61.7
**4d**	Tert-butylamino	5	35	52.3
**4e**	Dimethylamino	4	25	39.6
**4f**	Diethylamino	1.5	25	60.9
**4g**	Diisopropylamino	12	25	51.2
**4h**	Cyclohexylamino	8	40	41.8
**4i**	Pyrrolidinyl	1.5	40	42.1
**4j**	Morpholinyl	12	40	43.4

### 2.2. Antibacterial Activity

A preliminary antibacterial screeningof the synthesized compounds was performed using the disk diffusion method [53]. Table 2 lists the screening results of the tested compounds against the Gram negative bacteria *E. coli* (ATCC 25922) and *P. aeruginosa* (ATCC 27853) and Gram positive bacteria *S. aureus* (ATCC 25923) and *B. subtilis* (ATCC 6633). The results showed that most of the compounds showed antibacterial activities against the microorganisms at a dose of 1000 μg/mL. Compounds showing inhibition of at least 10 mm were considered active and were further evaluated for their minimal inhibitory concentration (MIC) [54,55].

**Table 2:** Inhibition zones (IZ) in mm (Mean ^a^ ± S.D ^b^) of the synthesized compounds **4a** to **4j**.

*Compounds*	*S. aureus*	*B. subtilis*	*E. coli*	*P. aeruginosa*
	*ATCC 25923*	*ATCC 6633*	*ATCC 25922*	ATCC 27853
**Apigenin**	11.1 ± 0.3	11.0 ± 1.0	10.1 ± 0.3	10.3 ± 0.2
**4a**	17.5 ± 0.1	21.3 ± 0.6	10.3 ± 0.1	8.6 ± 0.1
**4b**	17.9 ± 0.6	17.5 ± 0.4	10.9 ± 0.7	10.6 ± 0.2
**4c**	14.1 ± 0.5	15.2 ± 0.1	12.5 ± 0.1	10.5 ± 0.4
**4d**	14.8 ± 0.2	18.0 ± 0.3	12.8 ± 0.4	10.8 ± 0.1
**4e**	12.1 ± 0.6	15.9 ± 0.6	10.4 ± 0.2	12.8 ± 0.5
**4f**	18.3 ± 0.1	18.7 ± 0.5	14.8 ± 0.5	12.4 ± 0.3
**4g**	15.0 ± 0.3	19.0 ± 0.1	11.0 ± 0.3	10.0 ± 0.7
**4h**	22.1 ± 0.5	22.8 ± 0.2	15.3 ± 0.1	17.0 ± 1.0
**4i**	18.8 ± 0.4	19.3 ± 0.4	13.0 ± 0.1	10.2 ± 0.8
**4j**	25.9 ± 0.6	27.2 ± 0.8	15.6 ± 0.3	14.3 ± 1.0
**Ampicillin**	37.3 ± 0.6	36.7 ± 1.5	28.9 ± 0.1	33.7 ± 0.5
**Tetracycline**	26.3 ± 0.5	21.0 ± 1.0	21.7 ± 1.1	24.3 ± 1.1

^a^ Mean value of measured diameters of zones of inhibition; ^b^ S.D. denotes the standard deviation.

The MIC values of all the compounds are presented in Table 3. All the prepared apigenin analogs showed relatively higher antibacterial activities than apigenin. Gram-positive strains *S. aureus* and *B. subtilis* showed relatively high sensitivities toward the synthesized compounds, with MIC values from 1.95 μg/mL to 15.63 μg/mL. Furthermore, **4h** and **4j** showed prominent activities with MIC values of 3.91 and 1.95 μg/mL against *S. aureus* and *B. subtilis*, comparable with the positive control tetracycline, although lower than ampicillin. Compounds **4a**, **4b**, **4f**, and **4i** showed moderate activities against *S. aureus* and *B. subtilis* with MIC values of 7.81 μg/mL. However, **4c**–**e**, and **4g** exhibited weak antibacterial activities against *S. aureus* and *B. subtilis* with MIC values of 15.63 μg/mL to 31.25 μg/mL.

**Table 3:** *In vitro* antibacterial activity (MIC, μg/mL) and DPPH radical scavenging activity (IC_50_, μg/mL) of **4a** to **4j**.

*Compounds*	*IC_50_ (μg/mL) ^a^*	*MIC (μg/mL) ^a^*			
		*S. aureus*	*B. subtilis*	*E. coli*	*P. aeruginosa*
		ATCC 25923	ATCC 6633	ATCC 25922	ATCC 27853
**Apigenin**	1094.7 ± 2.2	31.25	31.25	62.5	62.5
**4a**	788.0 ± 1.6	7.81	3.91	62.5	125
**4b**	782.4 ± 2.3	7.81	7.81	62.5	62.5
**4c**	670.1 ± 1.5	15.63	15.63	31.25	62.5
**4d**	539.5 ± 1.4	15.63	7.81	31.25	62.5
**4e**	542.3 ± 1.3	31.25	15.63	62.5	31.25
**4f**	434.8 ± 1.4	7.81	7.81	15.63	31.25
**4g**	431.1 ± 1.1	15.63	7.81	62.5	62.5
**4h**	887.7 ± 2.5	3.91	3.91	15.63	15.63
**4i**	334.8 ± 1.2	7.81	7.81	31.25	62.5
**4j**	740.1 ± 1.8	1.95	1.95	15.63	15.63
**Ampicillin**	--	0.06	0.12	0.98	0.49
**Tetracycline**	--	1.95	3.91	3.91	3.91
**Vitamin C**	44.2 ± 1.1				

^a^ Average of three parallel experiments.

To analyze further the structure-activity relationships, the prepared apigenin analogs were divided into two categories. One category includes the cyclic substituent-containing apigenin analogs, **4h** to **4j**, and the other includes the aliphatic chain substituent-containing apigenin analogs, **4a** to **4g**. From Table 3, we find that the apigenin analogs showed relatively higher antibacterial activities than apigenin. Furthermore, in the cyclic substituent-containing apigenin analogs, the order of the MIC values was **4j** < **4h** < **4i**, indicating that the analogs with 6-membered rings had better antibacterial activities than those with 5-membered rings. Moreover, in the 6-membered ring compounds, compounds containing two heteroatoms (**4j**) showed stronger antibacterial activities than compounds containing one heteroatom (**4i**). For the aliphatic chain substituent-containing apigenin analogs, straight-chain primary amine-substituted apigenin analogs displayed stronger activities than the branched-chain substituted apigenin analogs.

### 2.3. Antioxidant Activity

**Figure 1:**
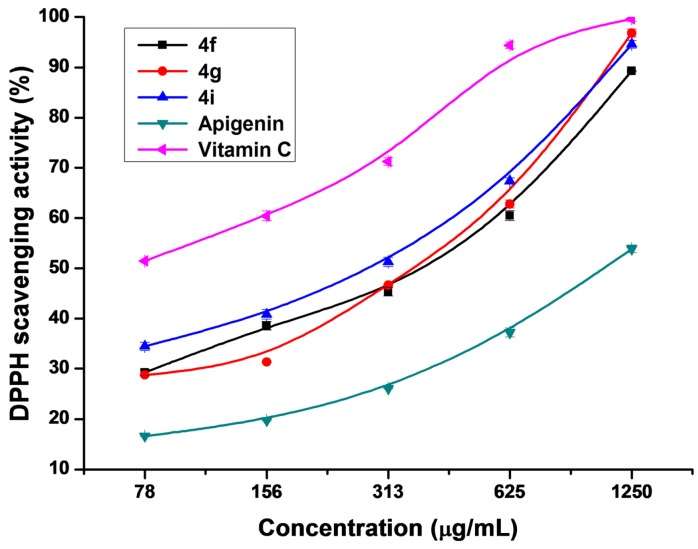
DPPH radical scavenging activities of **4f**, **4g**, **4i**, apigenin, and vitamin C.

The DPPH radical is a stable free radical commonly used as a substrate to evaluate *in vitro* antioxidant activities of extracts from fruits, vegetables, and medicinal plants [56]. An antioxidant can scavenge the radical by hydrogen donation, which results in a decrease in DPPH absorbance at 517 nm [57]. Generally, flavonoids display different DPPH radical scavenging activities with their different structures. The radical scavenging activities of flavonoids are highly controlled by the number and configuration of phenolic hydroxyl groups in the molecules and also influenced by the configuration of other substituents [58]. Table 3 shows the DPPH radical scavenging activities of the prepared apigenin analogs compared with those of the parent apigenin and vitamin C. Most of the prepared compounds exhibited potential scavenging activities compared with the parent apigenin and moderate activities compared with vitamin C. Compound **4i** was found to have significant antioxidant activity with the smallest IC_50_ value and compound **4h** possessed the least activity among these prepared compounds with the largest IC_50_ value. IC_50_ value is negatively related to antioxidant activity such that the lower the IC_50_ value is, the higher the antioxidant activity will be [59]. Therefore, the order of DPPH radical scavenging abilities of the analogs was **4i** > **4g** > **4f** > **4d** > **4e** > **4c** > **4j** > **4b** > **4a** > **4h** > apigenin. Compared with the parent apigenin, **4f**, **4g**, and **4i** exhibited significant antioxidant activities, with IC_50_ values of 434.8, 431.1, and 334.8 μg/mL, respectively. The concentration-dependent changes in the antioxidant activities of these compounds were also analyzed. The antioxidant activity was enhanced with the concentration of every sample was increased (Figure 1). When the concentration of **4f**, **4g**, and **4i** reached 1,250 μg/mL, their scavenging effects were close to that of vitamin C.

Apigenin analogs with branched-chain aliphatic substituents showed stronger antioxidant activities than those with straight-chain primary amine substituents or heteroatom-containing substituents, except for **4i**. Moreover, the alkyl number of the substituent groups determined the antioxidant activity of the analogs. The more alkyl numbers existed in the substituted apigenin analogs, the higher were the displayed activities, as shown by **4f** and **4g**. All these results above suggested that substituted apigenin analogs exhibited antioxidant activities due to the introduction of different electron donations on the C-8 of apigenin [58], and the antioxidant activities of flavonoids could be modulated by the number and configuration of amine groups in the molecules.

### 2.4. Antiproliferative Activity

**Figure 2:**
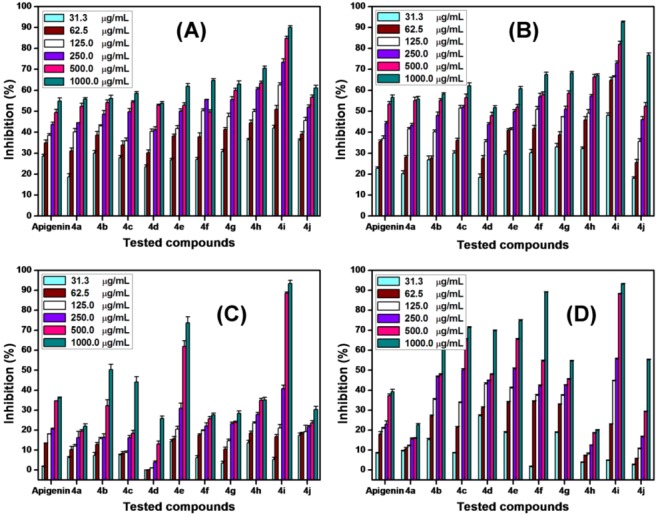
Inhibition ratios of apigenin and its analogs to the proliferation of HeLa (**A**), HepG2 (**B**), A549 (**C**), and MCF-7 (**D**) cell lines.

The antiproliferative activities of the prepared apigenin analogs **4a**–**j** were evaluated by performing *in vitro* assays of the inhibition ratios of these compounds to the proliferation of human cancer cells HeLa, HepG2, A549, and MCF-7. Figure 2 shows that the prepared apigenin analogs possessed different antiproliferative activities depending on the changes in their chemical structures, and high doses of these compounds showed more potent antiproliferative activities. Compound **4i** exhibited the most significant antiproliferative activity against the four cancer cells. At 1000 μg/mL, the inhibition rates of **4i** for HeLa, HepG2, A549, and MCF-7 cells were 89.97%, 92.60%, 93.33%, and 93.04%, respectively. All the prepared apigenin analogs possessed stronger inhibition effects on HeLa and HepG2 cells than on A549 and MCF-7 cells.

Table 4 summarizes the IC_50_ values of apigenin analogs against HeLa, HepG2, A549, and MCF-7 cells. Compound **4i** exhibited the strongest antiproliferative activity, and its IC_50_ values reached 40, 40, 223, and 166 μg/mL for HeLa, HepG2, A549, and MCF-7 cells, respectively. Moreover, compounds **4f**–**h**, and **4j** showed better antiproliferative activities (with lower IC_50_ values) against HeLa and HepG2 cells than apigenin, with inhibition ratios reaching more than 50% at 250 μg/mL. However, no obvious antiproliferative activity was observed for **4a**, **4b**, and **4d**, indicating the ineffectiveness of these compounds in suppressing the HeLa and HepG2 cell proliferation. Compounds **4b**, **4e**, and **4i** displayed stronger activities toward A549 cells, whereas **4e**, **4f**, and **4i** had better antiproliferative activities against MCF-7 cells, compared with apigenin. Moreover, the antiproliferative activities of the apigenin analogs against HeLa and HepG2 were enhanced with a short and more branched alkyl chain at the C-8 position of apigenin. The highest potency was obtained when a piperazin-1-yl-ethoxy group was present at the C-8 position of the apigenin. Combined with the structural features of the prepared analogs, the presence of nitrogen at the C-8 position of apigenin was critical to the antiproliferative activities of the apigenin analogs, which was supported by previous reports [60,61,62].

**Table 4:** Antiproliferative activities (IC_50_, μg/mL) of apigenin analogs against HeLa, HepG2, A549, and MCF-7 cell lines.

Compounds	IC_50_ (μg/mL)			
	HeLa	HepG2	A549	MCF-7
**1**	450 ± 2.0	460 ± 2.2	1740 ± 3.4	>2000 ± 3.6
**4a**	450 ± 2.5	470 ± 2,7	>2000 ± 4.3	>2000 ± 4.1
**4b**	430 ± 1.9	410 ± 2.2	1410 ± 3.0	420 ± 2.3
**4c**	270 ± 1.4	270 ± 1.9	>2000 ± 3.8	280 ± 1.7
**4d**	570 ± 2.0	620 ± 3.1	>2000 ± 4.0	310 ± 2.1
**4e**	230 ± 1.6	270 ± 2.0	380 ± 2.8	210 ± 1.9
**4f**	160 ± 1.5	150 ± 1.7	>2000 ± 3.7	250 ± 2.0
**4g**	210 ± 2.1	190 ± 1.5	>2000 ± 4.2	610 ± 3.2
**4h**	170 ± 1.7	180 ± 1.4	>2000 ± 3.5	>2000 ± 4.0
**4i**	40 ± 1.8	40 ± 1.5	223 ± 2.1	166 ± 1.8
**4j**	180 ± 2.0	220 ± 2.0	>2000 ± 4.4	1010 ± 2.9

## 3. Experimental

### 3.1. General

Apigenin (>98%) was purchased from Xi’an Spring-chem Bio-tech Co., Ltd. (Xi’an, China). Tetracycline and DPPH were from Sigma-Aldrich (St. Louis, MO, USA). Ampicillin and MTT were obtained from Amersco Inc. (Solon, OH, USA). Yeast extract and tryptone were from Oxoid Ltd. (Basingstoke, Hampshire, UK). Dulbecco’s modified eagle medium, RPMI-1640 medium, and fetal bovine serum (FBS) were from Gibco Invitrogen Corporation (Carlsbad, CA, USA). HeLa and HepG2 cells were provided by the Cell Center of the Fourth Military Medical University (Xi’an, China). A549 and MCF-7 cells were from the Chinese Academy of Sciences (Shanghai, China). Vitamin C and primary or secondary amines were from Aladdin (Shanghai, China). *N*,*N*-Dimethylformamide (DMF) was purified by vacuum distillation over CaH_2_ before utilization. All solvents and other chemicals were supplied by local commercial suppliers and of analytical reagent grade, unless otherwise stated. Deionized water (Milli-Q, Millipore, Bedford, MA, USA) was used to prepare aqueous solutions. Column chromatography was performed with silica gel 60 (200 to 300 mesh). Thin-layer chromatography (TLC) and preparative TLC (PTLC) were used with silica gel 60 GF254 (Qingdao Haiyang Chem. Co., Ltd., Shandong, China). Product spots were detected under UV light using ZF-6 type III ultraviolet analyzer (Shanghai Jiapeng Technology Co., Ltd., Shanghai, China). Melting points were determined using a digital melting point apparatus (Zhengzhou Mingze Technology Co., Ltd., Zhengzhou, China) and were uncorrected. ESI mass spectra were obtained using a Thermo Scientific LCQ FLEET mass spectrometer equipped with an electrospray ion source and controlled by Xcalibur software (Thermo Fisher Scientific, Waltham, MA, USA). Proton nuclear magnetic resonance spectra (^1^H-NMR) and carbon-13 nuclear magnetic resonance spectra (^13^C-NMR) were obtained using a Bruker Avance DMX 500 MHz/125 MHz spectrometer (Bruker, Billerica, MA, USA), with chemical shifts reported in parts per million [in dimethyl sulfoxide (DMSO-*d*_6_), tetramethylsilane as an internal standard, *J* values were given in Hertz]. MIC was detected using a BIO-RAD 680 Microplate reader (Beijing Yuanye Bio. Co., Ltd., Beijing, China). The absorbance values in DPPH assays were recorded using a UV-1600PC spectrophotometer (Shanghai Mapada Instruments Co., Ltd., Shanghai, China).

### 3.2. General Procedure for the Synthesis of Compounds ***4a*** to ***4j***

The apigenin analogs **4a**–**j** were synthesized via Mannich reactions [63]. A magnetic stirring bar and apigenin (**1**, 100 mg, 0.37 mmol) dissolved in anhydrous DMF (1 mL) were first placed into a two-necked flask. Then, methanol (15 mL) was added and a yellow solution was immediately obtained. The reaction system was then immersed in an oil bath while being stirred. Then, formaldehyde solution (0.028 mL, 37%) and ethylamine solution (0.031 mL, 70%) were added dropwise to the reaction mixture. Stirring was required to keep the reaction mixture homogenized during these operations. The reaction process was checked using TLC analysis. After 4 h reaction, the mixture was filtered, and the supernatant was collected. The solvent was removed under reduced pressure. The residue was then purified using PTLC with ethyl acetate-methanol (1:1, v/v) as developing agent to obtain **4a**. Compounds **4b** to **4j** were prepared using similar methods, and their structures were confirmed via melting point determination, ^1^H-NMR, ^13^C-NMR, and ESI-MS. The data are listed as follows.

*4',5,7-Trihydroxy-8-(ethylamino)-2-phenyl-4H-chromen-4-one* (**4a**): yellow solid, yield 40.5%, m.p. >300 °C. ^1^H-NMR δ: 13.00 (s, 1H, 5-OH), 7.84 (d, 2H, *J* = 10.0 Hz), 6.92 (d, 2H, *J* = 10.0 Hz), 6.59 (s, 1H), 6.00 (s, 0.6H), 5.77 (s, 0.4H), 4.14 (s, 0.9H, CH_2_), 3.96 (s, 1.1H, CH_2_), 2.81 (m, 2H, -NH-CH_2_-CH_3_), 1.29 (t, 3H, -NH-CH_2_-CH_3_); ^13^C-NMR δ: 182.0, 169.9, 163.5, 162.0, 159.6, 159.2, 128.4, 128.4, 121.7, 116.6, 116.6, 103.1, 102.7, 102.3, 95.7, 41.7, 35.7, 17.6; ESI-MS (*m/z*): 325.9 [M−H]^−^.

*4',5,7-Trihydroxy-8-(propylamino)-2-phenyl-4H-chromen-4-one* (**4b**): yellow solid, yield 38.7%, m.p. >300 °C. ^1^H-NMR δ: 12.99 (s, 1H, 5-OH), 7.85 (d, 2H, *J* = 10.0 Hz), 6.90 (d, 2H, *J* = 10.0 Hz), 6.60 (s, 0.3H), 6.56 (s. 0.7H), 6.02 (s, 0.6H), 5.79 (s, 0.4H), 4.14 (s, 0.7H, CH_2_), 3.95 (s, 1.3H, CH_2_), 2.71 (t, *J* = 19.1 Hz, 2H, -NH-CH_2_-CH_2_-CH_3_), 1.54–1.58 (m, 2H, -NH-CH_2_-CH_2_-CH_3_), 0.91 (t, *J* = 6.7 Hz, 3H, -NH-CH_2_-CH_2_-CH_3_); 13C-NMR δ: 181.8, 169.9, 163.4, 161.5, 159.0, 157.0, 128.8, 128.8, 122.4, 116.7, 116.7, 103.7, 102.8, 102.1, 95.9, 42.3, 36.1, 27.5, 15.9; ESI-MS (*m/z*): 339.9 [M−H]^−^.

*4',5,7-Trihydroxy-8-(isopropylamino)-2-phenyl-4H-chromen-4-one* (**4c**): yellow solid, yield 61.7%, m.p. >300 °C. ^1^H-NMR δ: 12.99 (s, 1H, 5-OH), 7.86 (d, 2H, *J* = 10.0 Hz), 6.91 (d, 2H, *J* = 10.0 Hz), 6.59 (s, 0.6H), 6.56 (s, 0.4H), 6.01 (s, 0.4H), 5.79 (s, 0.6H), 4.14 (s, 1.1H, CH_2_), 3.96 (s, 0.9H, CH_2_), 3.07–3.14 (m, 1H, -NH-CH(CH_3_)_2_), 1.19 (d, 6H, -NH-CH(CH_3_)_2_); ^13^C-NMR δ: 181.9, 168.2, 163.4, 158.9, 158.0, 155.4, 128.4, 128.4, 121.1, 116.3, 116.3, 102.7, 102.4, 100.0, 95.8, 48.4, 40.4, 20.3; ESI-MS (*m/z*): 340.0 [M−H]^−^.

*4',5,7-Trihydroxy-8-(tert-butylamino)-2-phenyl-4H-chromen-4-one* (**4d**): yellow solid, yield 52.3%, m.p. >300 °C. ^1^H-NMR δ: 12.98 (s, 1H, 5-OH), 7.84 (d, 2H, *J* = 10.0 Hz), 6.92 (d, 2H, *J* = 10.0 Hz), 6.57 (s, 0.4H), 6.54 (s, 0.6H), 6.00 (s, 0.6H), 5.77 (s, 0.4H), 4.14 (s, 1.1H, CH_2_), 3.96 (s, 0.9H, CH_2_), 1.27 (s, 9H, (CH_3_)_3_C-); ^13^C-NMR δ: 180.8, 168.7, 162.9, 161.5, 158.0, 155.4, 128.6, 128.6, 125.6, 116.7, 116.7, 102.6, 102.4, 101.5, 95.9, 54.0, 49.1, 26.6; ESI-MS (*m/z*): 353.9 [M−H]^−^.

*4',5,7-Trihydroxy-8-(dimethylamino)-2-phenyl-4H-chromen-4-one* (**4e**): yellow solid, yield 39.6%, m.p. >300 °C. ^1^H-NMR δ: 13.03 (s, 1H, 5-OH), 7.94 (d, 2H, *J* = 10.0 Hz), 6.96 (d, 2H, *J* = 10.0 Hz), 6.77 (s, 1H), 6.11 (s, 1H), 3.97 (s, 2H, CH_2_), 2.43 (s, 6H, -N(CH_3_)_2_); ^13^C-NMR δ: 181.9, 168.2, 163.4, 161.5, 161.1, 155.4, 128.8, 128.8, 121.9, 116.5, 116.5, 103.0, 102.7, 100.0, 99.9, 53.4, 43.9; ESI-MS (*m/z*): 325.8 [M−H]^−^.

*4',5,7-Trihydroxy-8-(diethylamino)-2-phenyl-4H-chromen-4-one* (**4f**): yellow solid, yield 60.9%, m.p. >300 °C. ^1^H-NMR δ: 13.47 (s, 1H, 5-OH), 7.89 (d, 2H, *J* = 8.0 Hz), 6.92 (d, 2H, *J* = 8.0 Hz), 6.71 (s, 1H), 6.29 (s, 1H), 3.92 (s, 2H, CH2), 2.75 (q, 4H, *J* = 10.0Hz, -N(CH_2_CH_3_)_2_), 1.11 (t, 6H, *J* = 10.0 Hz, -N(CH_2_CH_3_)_2_; ^13^C-NMR δ: 181.8, 169.3, 163.6, 161.6, 159.0, 157.2, 128.7, 128.7, 121.8, 116.4, 116.4, 103.6, 102.9, 102.2, 94.9, 48.5, 46.3, 10.7; ESI-MS (*m/z*): 354.0 [M−H]^−^.

*4',5,7-Trihydroxy-8-(diisopropylamino)-2-phenyl-4H-chromen-4-one* (**4g**): yellow solid, yield 51.2%, m.p. >300 °C. ^1^H-NMR δ: 13.45 (s, 1H, 5-OH), 10.37 (s, 1H, 7-OH), 7.91 (d, 2H, *J* = 10.0 Hz), 6.93 (d, 2H, *J* = 10.0 Hz), 6.72 (s, 1H), 6.25 (s, 1H), 3.96 (s, 2H, CH_2_), 3.18–3.25 (m, 2H,-N(CH(CH_3_)_2_)_2_, 1.15(d, 12H, *J* = 5.0 Hz, -N(CH(CH_3_)_2_)_2_; ^13^C-NMR δ: 181.8, 169.9, 163.6, 161.5, 158.3, 157.0, 128.8, 128.8, 121.9, 116.4, 116.4, 104.0, 103.0, 102.1, 94.9, 49.5, 41.9, 19.2; ESI-MS (*m/z*): 381.8 [M−H]^−^.

*4',5,7-Trihydroxy-8-(cyclohexylamino)-2-phenyl-4H-chromen-4-one* (**4h**): yellow solid, yield 41.8%, m.p. >300 °C. ^1^H-NMR δ: 13.01 (1H, s, 5-OH), 7.85 (d, 2H, *J* = 10.0 Hz), 6.89 (d, 2H, *J* = 10.0 Hz), 6.58 (s, 0.6H), 6.55 (s, 0.4H), 6.04 (s, 0.4H), 5.80 (s, 0.6H), 4.15 (s, 1.2H, CH_2_), 3.97 (s, 0.8H, CH_2_), 2.74 (s, 1H, cyclohexylamine-CH), 1.24–1.97 (m, 10H, cyclohexylamine-CH_2_); ^13^C-NMR δ: 181.0, 169.0, 162.8, 162.6, 161.1, 158.7, 128.5, 128.5, 121.2, 116.7, 116.7, 102.4, 101.0, 100.7, 95.5, 55.5, 55.2, 31.2, 25.7, 24.5; ESI-MS (*m/z*): 379.9 [M−H]^−^.

*4',5,7-Trihydroxy-8-(pyrrolidin-1-ylmethyl)-2-phenyl-4H-chromen-4-one* (**4i**): yellow solid, yield 42.1%, m.p. >300 °C. ^1^H-NMR δ: 13.53 (1H, s, 5-OH), 7.89 (d, 2H, *J* = 5.0 Hz), 6.91 (d, 2H, *J* = 5.0 Hz), 6.69 (s, 1H), 6.28 (s, 1H), 3.95(s, 2H, CH2), 2.80 (s, 4H, pyrrolidine-N(CH_2_)_2_), 1.83 (s, 4H, pyrrolidine-(CH_2_)_2_); ^13^C-NMR δ: 181.5, 170.1, 163.4, 161.7, 159.0, 157.4, 128.7, 128.7, 121.7, 116.5, 116.5, 104.6, 102.7, 101.5, 95.0, 53.2, 49.9, 23.6; ESI-MS (*m/z*): 351.9 [M−H]^−^.

*4',5,7-Trihydroxy-8-(morpholinomethyl)-2-phenyl-4H-chromen-4-one* (**4j**): yellow solid, yield 43.4%, m.p. >300 °C. ^1^H-NMR δ: 12.98 (s, 1H, 5-OH), 8.03 (d, 2H, *J* = 5.0 Hz), 6.91 (d, 2H, *J* = 5.0 Hz), 6.83 (s, 1H), 6.58 (s, 1H), 4.11 (s, 2H, CH2), 3.81 (s, 2H, morpholine-O-CH_2_), 3.75 (s, 2H, morpholine-O-CH_2_), 2.52 (s, 4H, morpholine-N(CH_2_)_2_); ^13^C-NMR δ: 182.5, 165.0, 164.0, 161.9, 160.7, 154.9, 128.9, 128.9, 121.7, 116.6, 116.6, 104.3, 103.3, 102.9, 101.5, 66.6, 53.3, 50.9; ESI-MS (*m/z*): 368.0 [M−H]^−^.

### 3.3. Antibacterial Assay

The bacterial strains were kept in liquid nitrogen (−196 °C) in a Luria-Broth (LB) medium (5 g/L yeast extract, 10 g/L bactopeptone, and 10 g/L sodium chloride) containing 15% glycerol. Prior to the experiment, the bacterial strains were grown on LB agar plates at 37 °C. A single colony of bacteria from an overnight culture was inoculated into 150 mL of fresh LB medium. Each bacterial colony was then continuously incubated for 12 h at 37 °C in a rotary shaker set to 200 rpm. The cell density was determined via the normal plate count method [64]. The inocula were then diluted with 0.9% sterile normal saline to obtain a cell density of approximately 1.5 × 10^8^ CFU/mL to match the 0.5 McFarland standard [65].

Antibacterial activity was done by the disk diffusion method according to the National Committee for Clinical Laboratory Standards (NCCLS) [66]. Ten μL of bacterial suspension was mixed with sterile LB agar medium (10 mL) at 40 °C and poured onto an agar plate. Sterile paper discs (6.0 mm in diameter) impregnated with compound dissolved in dimethylsulfoxide (DMSO) at concentrations of 1000 μg/mL were prepared. Then, the impregnated paper discs were placed on the surface of the media inoculated with the microorganism. Ampicillin and tetracycline were used as positive controls. DMSO poured disk was used as negative control. The diameter of zone of growth inhibition around the disc was measured after 18 h of incubation at 37 °C. An average of three independent determinations was recorded.

The MICs for the synthesized compounds were also determined using the National Committee for Clinical Laboratory Standards in a LB medium in 96-well tissue culture plates [67]. All the tested compounds were dissolved in DMSO and diluted in the growth media, resulting in the final concentration ranging from 0.06 μg/mL to 500 μg/mL. Afterward, 100 μL of this solution was pipetted into the first well of each line in the 96-well tissue culture plate, which contained 100 μL of the LB medium. The solution was then serially diluted to obtain two-fold serial dilutions of the test compounds and positive controls in the subsequent wells, which contained 100 μL LB medium. The 0.5 McFarland-matched bacterial suspension (1 mL) was then diluted with 100 mL of the medium. The diluted bacterial suspension (100 μL) was added to each well and then kept for incubation. One well containing bacterial cells and DMSO without any test compounds (growth control), and one well containing only growth medium (sterility control), were used as controls. Ampicillin and tetracycline were used as positive controls. The maximum concentration of the test compounds was 125 μg/mL. The sealed microtiter plates were incubated at 37 °C in a moist, dark chamber. MIC values were recorded spectrophotometrically at 630 nm after 24 h incubation. All compounds were tested in triplicate. The experiments were repeated at least thrice.

### 3.4. Antioxidant Assay

DPPH free radical scavenging activity was measured to evaluate the antioxidant activity via the previously reported method [68]. The chemical compounds were dissolved in methanol to obtain a final concentration ranging from 78 μg/mL to 1,250 μg/mL to determine IC_50_ (a high concentration results in 50% inhibition of DPPH color). A methanol solution of DPPH (0.2 mM) was incubated with a methanol solution of each of the test samples (78 μg/mL to 1250 μg/mL) for 30 min at room temperature (25 °C) in the dark. The DPPH radical scavenging activity was determined by measuring the absorbance at 517 nm using a spectrophotometer. The DPPH radical scavenging activity of Vitamin C was also assayed for control, whereas that of methanol was used as a blank. The percentage of DPPH radical scavenging effect was calculated as follows:

[(*A_control_* − *A_test_*)/*A_control_*] × 100%

where *A_control_* is the absorbance of the control (DPPH solution without any test sample) and *A_test_* is the absorbance of the test sample (DPPH solution plus test sample). All tests were performed in triplicate.

### 3.5. Antiproliferative Assay

The inhibition effects of **4a**–**j** on the HeLa, HepG2, A549, and MCF-7 cell proliferation were evaluated *in vitro* via MTT staining according to the procedures reported previously [69,70], with slight modification. The four selected cancer cell lines, HeLa, HepG2, A549, and MCF-7 were routinely cultured using suitable medium supplemented with 10% FBS, 100 U/mL penicillin, and 100 μg/mL streptomycin at 37 °C and 5% CO_2_ with 95% humidity. The cells were normally passaged at a ratio of 1:3 every 3 days to maintain them in the exponential growth phase. Before use, the cells were harvested through trypsinization with 0.25% trypsin in Ca^2+^- and Mg^2+^-free Hanks’ balanced salt solution at 37 °C. Trypsinization was stopped through the addition of fresh supplemented medium. The cell suspension was centrifuged at 1000 rpm for 5 min. The cells were then resuspended in supplemented medium (1.0 × 10^6^ cells/well in 6-well plates) for further use. Then, the cells were seeded into 96-well microtiter plates (4.0 × 10^3^ cells per well) with fresh medium (150 μL). After 24 h incubation, the tested compounds (150 μL, final concentrations of 62.5, 125, 250, 500, 1000, or 2000 μg/mL in the culture medium) were added to each well and continuously cultured for another 24 h. Afterward, MTT (20 μL, 5 mg/mL) was added to each well, which was then cultured for another 4 h under similar conditions. Finally, DMSO (150 μL) was added to terminate the reaction. The survival rate of the cancer cells was evaluated by measuring the optical density (*A*) on a microplate reader (model 680, BIO-RAD, Hercules, CA, USA) at 490 nm. All *in vitro* results were expressed as the cancer cell proliferation inhibition ratio according to the formula below:

[(*A_control_* − *A_test_*)/*A_control_*] × 100%

where *A_control_* and *A_test_* are the optical densities of the control and the test groups, respectively. All assays were done in triplicate.

## 4. Conclusions

In summary, we have synthesized a series of 8-aminomethylated apigenin analogs. Bioactivity assays showed that all the synthesized compounds exhibited greater potential antiproliferative, antibacterial, and antioxidant activities compared with the parent apigenin. Among these apigenin analogues, compound **4j** was found to be the most active, with MIC values of 1.95, 1.95, 15.63, and 15.63 μg/mL against *S. aureus*, *B. subtilis*, *E. coli*, and *P. aeruginosa*, respectively. Compound **4i** exhibited the strongest antiproliferative activities against HeLa, HepG2, A549, and MCF-7 cells. Compound **4i** may also act as an antioxidant agent because of its potent DPPH scavenging activity. 

Previous reports [49,50,51,60,61] have shown that the bioactivity increased when aminomethyl groups were incorporated at the C-8 position. Our results are consistent with this. The above screening results also showed that the increased biological activities were due to the introduction of aminomethyl groups into the C-8 position of the parent apigenin. However, further pharmaceutical studies on the mechanisms of the antiproliferative, antibacterial, and antioxidant activities of the apigenin analogs are still necessary. In addition, the synthesis yields of these apigenin analogs should be improved. All these studies are currently under way in our laboratory.
